# A novel mechanism of post-translational modulation of HMGA functions by the histone chaperone nucleophosmin

**DOI:** 10.1038/srep08552

**Published:** 2015-02-25

**Authors:** Laura Arnoldo, Riccardo Sgarra, Eusebio Chiefari, Stefania Iiritano, Biagio Arcidiacono, Silvia Pegoraro, Ilenia Pellarin, Antonio Brunetti, Guidalberto Manfioletti

**Affiliations:** 1 Department of Life Sciences, University of Trieste, Trieste, 34127, Italy; 2 Department of Health Sciences, University "Magna Græcia" of Catanzaro, Catanzaro, 88100, Italy

## Abstract

High Mobility Group A are non-histone nuclear proteins that regulate chromatin plasticity and accessibility, playing an important role both in physiology and pathology. Their activity is controlled by transcriptional, post-transcriptional, and post-translational mechanisms. In this study we provide evidence for a novel modulatory mechanism for HMGA functions. We show that HMGAs are complexed *in vivo* with the histone chaperone nucleophosmin (NPM1), that this interaction requires the histone-binding domain of NPM1, and that NPM1 modulates both DNA-binding affinity and specificity of HMGAs. By focusing on two human genes whose expression is directly regulated by HMGA1, the Insulin receptor (*INSR*) and the Insulin-like growth factor-binding protein 1 (*IGFBP1*) genes, we demonstrated that occupancy of their promoters by HMGA1 was NPM1-dependent, reflecting a mechanism in which the activity of these *cis*-regulatory elements is directly modulated by NPM1 leading to changes in gene expression. HMGAs need short stretches of AT-rich nucleosome-free regions to bind to DNA. Therefore, many putative HMGA binding sites are present within the genome. Our findings indicate that NPM1, by exerting a chaperoning activity towards HMGAs, may act as a master regulator in the control of DNA occupancy by these proteins and hence in HMGA-mediated gene expression.

Chromatin accessibility is a main determinant in activating gene transcription. Besides histones, non-histone chromatin proteins are among the main actors involved in regulating chromatin plasticity. An important group of non-histone proteins is constituted by the high mobility group A (HMGA) family of architectural transcription factors, which includes HMGA1a and HMGA1b protein isoforms, derived from alternative splicing of the *HMGA1* mRNA, and HMGA2, which is encoded by the separate but highly related gene, *HMGA2*^1^. HMGAs are considered oncofetal proteins, since they are highly expressed and play essential roles during embryonic development and tumorigenesis. Although their expression is low in many adult tissues^1^, HMGA1 is required for proper transcription of the insulin receptor (*INSR*) gene in both differentiated insulin target cells in culture and in insulin target tissues from humans and adult mice^2^,^3^. Furthermore, we recently showed that HMGA1, as a downstream target of the INSR signaling pathway, plays a key role in the nutritionally and insulin-regulated transcription of genes involved in glucose metabolism, such as the *IGFBP1* gene, as well as the gluconeogenic genes *PEPCK* and *G6Pase*^4^.

HMGA proteins have no intrinsic transcriptional activity per se; rather, they have been shown to transactivate promoters through mechanisms that facilitate the assembly and stability of stereospecific DNA-protein complexes so-called “enhanceosomes”, which promote gene transcription in response to extracellular and intracellular signals. HMGAs perform this task by modifying DNA conformation and by recruiting transcription factors to specific promoter regions, favouring DNA-protein and protein-protein interactions^5^,^6^, thus governing the expression of an impressive number of mammalian genes^7^,^8^,^9^. Despite the fact that the mechanism(s) of their regulation is still not completely known, HMGAs are finely modulated at different levels. In fact, in addition to transcriptional regulation^10^,^11^,^12^, HMGA expression is post-transcriptionally regulated by miRNAs^13^,^14^,^15^,^16^ and pseudogenes^17^, while their activity is under the surveillance of a series of post-translational modifications^18^.

Using a protein-protein interaction screening system, we previously identified three novel HMGA molecular partners: the nuclear chaperones nucleophosmin (NPM1), nucleolin (NCL), and the histone chromatin assembly factor 1 (CAF-1)^19^,^20^,^21^. Here we demonstrate that binding of NPM1 to HMGA proteins efficiently modulates HMGA's DNA-binding affinity and specificity towards two target gene promoters, the *INSR* and the *IGFBP1* promoters, whose functions are implicated in glucose metabolism and the maintenance of glucose homeostasis. To our knowledge, our results in the present work demonstrate a novel regulatory mechanism for the activity of HMGA proteins, which is mediated by NPM1.

## Results

### NPM1 interacts *in vivo* with HMGA proteins

**Figure 1:**
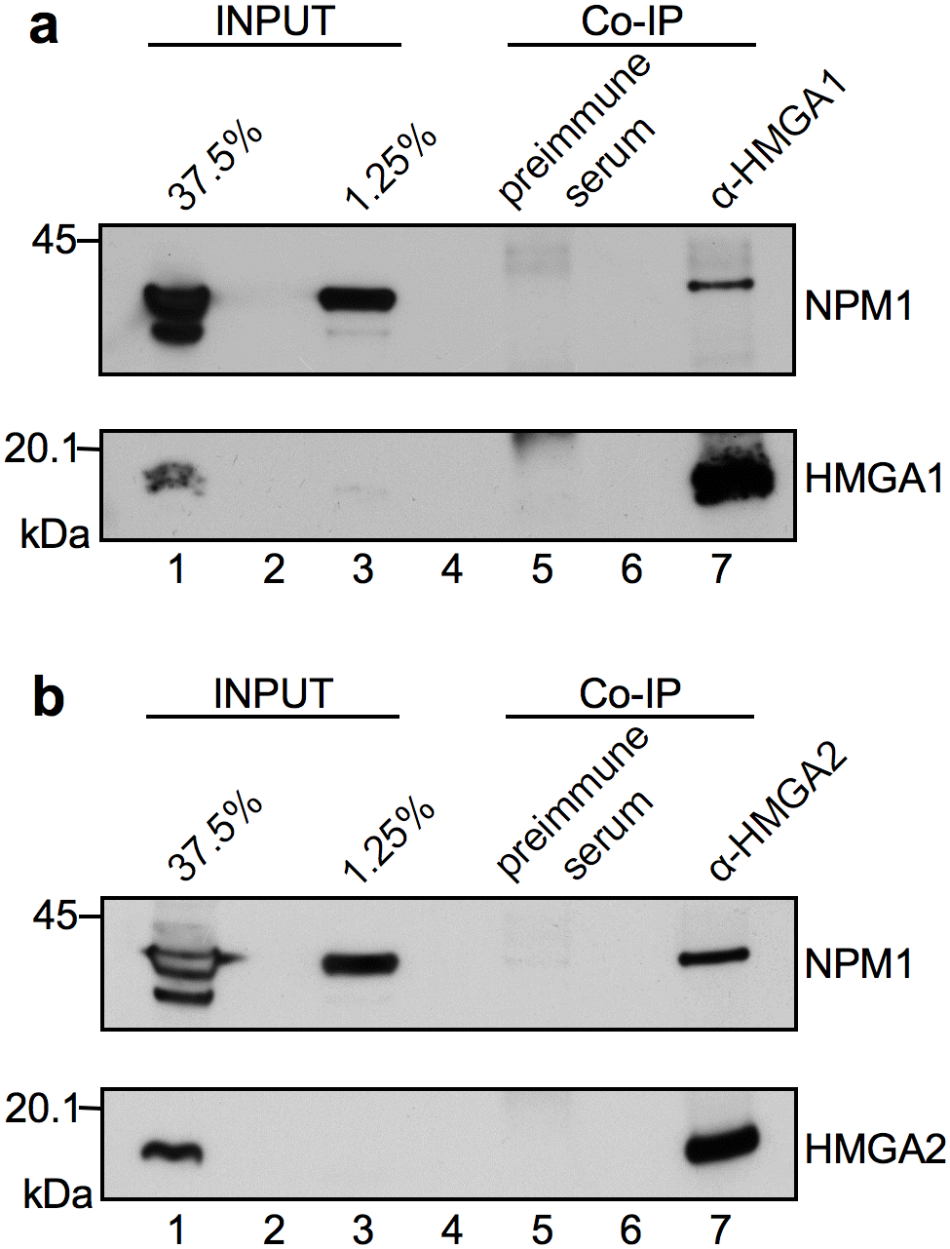
HMGAs and NPM1 associate *in vivo*. (a) Co-IP experiments performed with cellular lysates from HepG2 cells. Protein lysate was immunoprecipitated with α-HMGA1 specific antibody and NPM1 was detected by western-blot using an α-NPM1 antibody (lane 7). Co-IP performed with a pre-immune serum was included as a control of specificity (lane 5). Lanes 1 and 3: inputs to check the presence of HMGA1 and NPM1 in cell lysates. (b) Co-IP was performed for HMGA2 protein as described for panel a, using an α-HMGA2 specific antibody.

Using an affinity chromatography-based proteomic approach, we previously reported an *in vitro* interaction between HMGAs and NPM1^19^. To test whether these factors can also associate *in vivo*, co-immunoprecipitation assays were performed. HepG2 cell lysates were immunoprecipitated with α-HMGA1 and α-HMGA2 antibodies and the immunocomplexes analyzed by western-blotting with an α-NPM1 antibody. As shown in Fig. 1a, NPM1 associates with HMGA1 (lane 7), while complex formation was not detected in the negative control (lane 5 – pre-immune serum). As a control, cell lysates were tested for the expression of HMGA1 and NPM1 proteins; two different amounts of lysate were used given the different signals obtained for HMGA1 and NPM1 (lanes 1 and 3). The same approach has been used to demonstrate the *in vivo* interaction between HMGA2 and NPM1 (Fig. 1b).

NPM1 is generally considered a nucleolar protein; however, in accordance with literature data^24^, immunofluorescence analyses clearly indicate that, despite its predominant localization in nucleoli, NPM1 has also a nucleoplasmic localization in HepG2 cells (Supplementary Fig. S1). Therefore, these experiments clearly demonstrate that endogenous HMGA and NPM1 proteins share the same nuclear sub-compartment and interact each other *in vivo*.

### The central acidic clusters of NPM1 bind the basic protein-protein interaction domain of HMGAs

**Figure 2:**
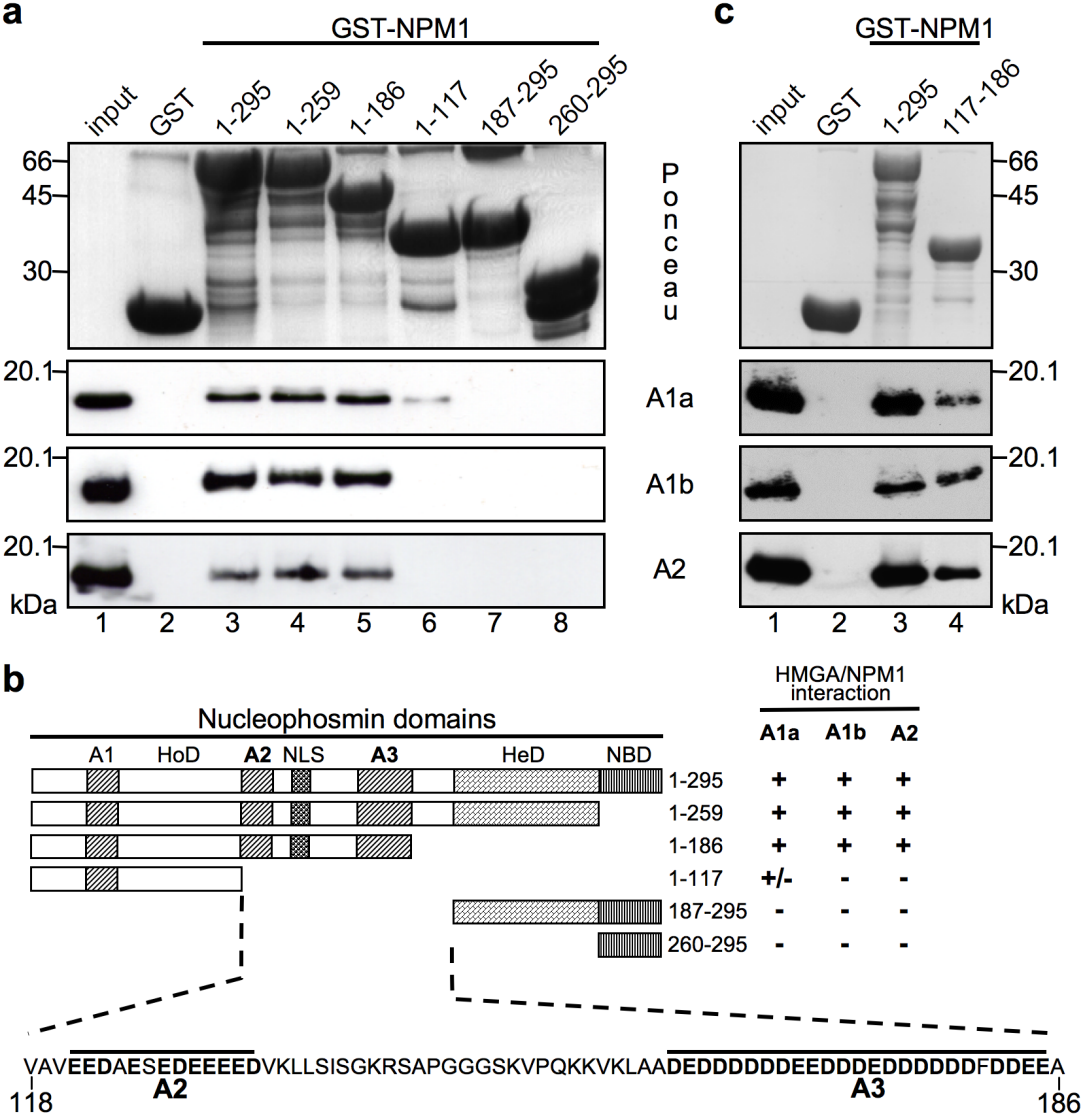
The NPM1 central region, containing two acidic clusters, mediates the interaction with HMGAs. (a) Protein-protein interaction mapping experiment performed with GST pull-down assays using GST-fused N- and C-terminal truncated NPM1 forms and recombinant HMGA proteins (HMGA1a: A1a; HMGA1b: A1b; HMGA2: A2). Bound proteins were visualized by western-blot using α-HMGA1 and α-HMGA2 antibodies. GST was used as a negative control. (b) Schematic representation of protein-protein interaction mapping. The different NPM1 deletion mutants used and the amino acid sequence of the HMGA-interacting region are reported. A1, A2, and A3: acidic regions. HoD and HeD: homo- and hetero-dimerization domain, respectively. NLS: nuclear localization signal. NBD: Nucleotide binding domain. (c) Protein-protein interaction mapping experiment performed with GST pull-down assay, using GST-fused NPM1 full length (1–295) and NPM1 117–186 with recombinant HMGA proteins. Bound proteins were visualized by western-blot using α-HMGA1 and α-HMGA2 antibodies. GST was used as a negative control. Representative red ponceau stained membranes are shown as quantity and integrity control of the GST fusion proteins used.

Several functional domains have been mapped within NPM1 aminoacidic sequence^25^,^26^: the N-terminus contains an oligomerization domain (HoD); the central portion has two highly acidic stretches (A2 and A3) separated by a nuclear localization signal (NLS), whereas the C-terminal portion is formed by a heterodimerization domain (HeD) mediating interactions with other nuclear proteins and a nucleic acid-binding domain (NBD) (Fig. 2). We mapped the NPM1 interaction domain using a series of N- and C-terminal GST-fused NPM1 deletion mutants in pull-down experiments with recombinant HMGA1a, HMGA1b, and HMGA2 proteins (Fig. 2a). Results summarized in Fig. 2b clearly show that NPM1/HMGAs interaction is abrogated when the central portion of NPM1 is deleted (amino acid residues 118–186). In order to assess whether this region itself binds HMGAs, we performed GST pull-down with GST-NPM1 117-186 and HMGA proteins (Fig. 2c). Results show that, albeit with lower affinity with respect to the full-length protein, the central portion of NPM1 containing the two acidic clusters (A2 and A3 – Fig. 2b) is sufficient to interact with HMGA proteins. Interestingly, these two clusters are responsible for the histone chaperone activity of NPM1^27^. Moreover, GST pull-down experiments, using HA-tagged full length HMGA proteins, allowed us to perform a quantitative comparison using the same antibody to detect both proteins (HA-HMGA1a and HA-HMGA2), and to determine that NPM1 displays almost the same affinity towards the different HMGA proteins (supplementary Fig. S2).

**Figure 3:**
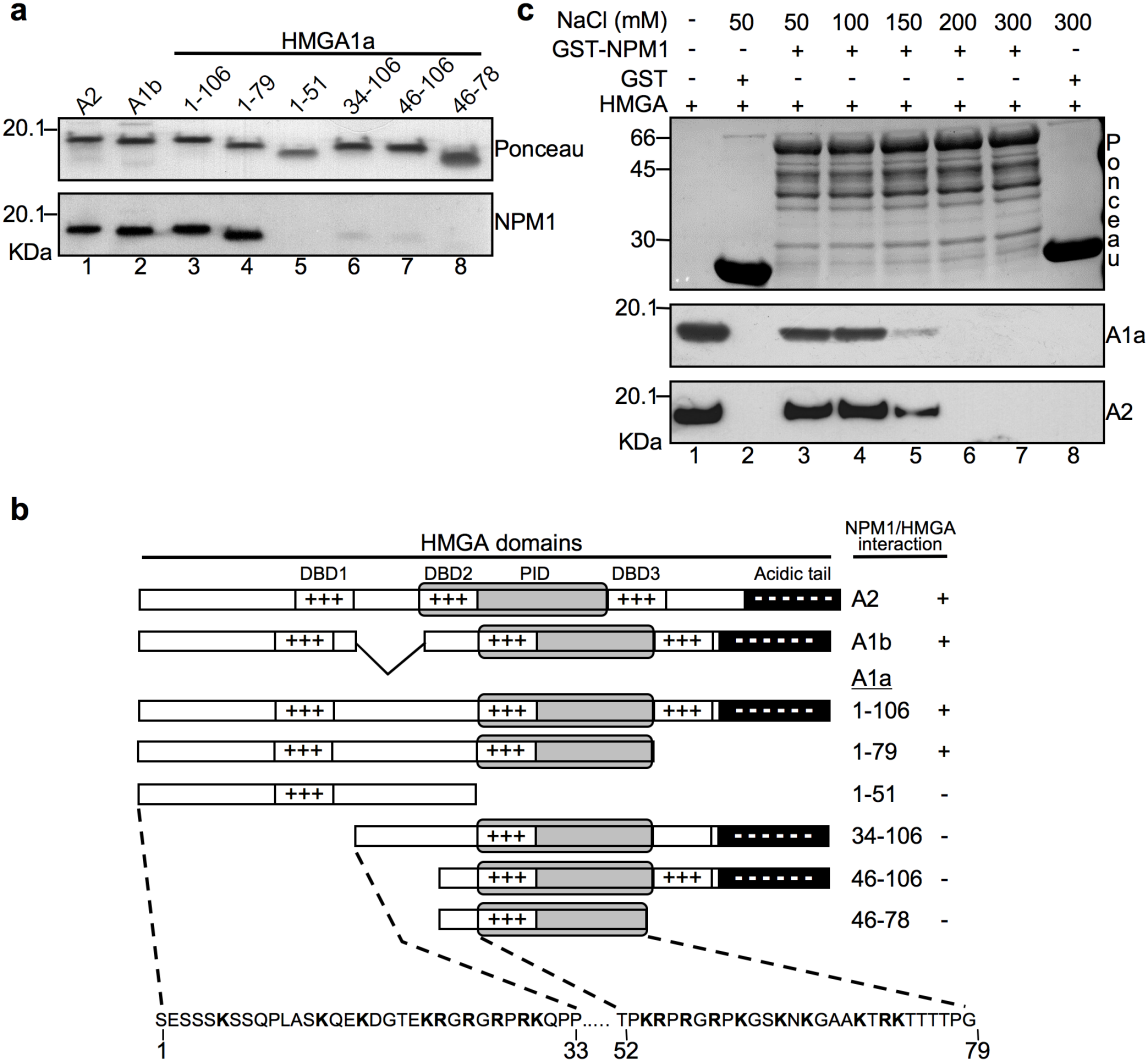
Multiple HMGA aminoacidic regions are involved in NPM1 binding. (a) Equivalent amounts of HMGA2, HMGA1b, HMGA1a, and HMGA1a truncated forms were SDS-PAGE separated, blotted, and red ponceau stained. Membrane was then subjected to farwestern analysis. *In vitro* transcribed, translated, and [^35^S]-radiolabelled full length NPM1 (1-295) was used as a probe. Interacting proteins were detected by fluorography. (b) Schematic representation of farwestern results. HMGA forms used are schematically visualized and the aminoacid sequence of the NPM1-interacting region is reported. DBD: DNA binding domain. PID: protein/protein interaction domain. Acidic tail: C-terminal acidic tail. (c) GST pull-down experiments were performed with full length GST-fused NPM1 and recombinant HMGA1a (A1a) and HMGA2 (A2) proteins at increasing ionic strength conditions from 50 to 300 mM NaCl (lanes 3-7). GST was incubated with HMGA proteins at low or high (50 and 300 mM NaCl) ionic strength conditions as a control (lanes 2 and 8). Equivalent amounts of HMGA proteins used in the GST pull-down experiments were loaded as references (lane 1). Bound proteins were visualized by western-blot using α-HMGA1 and α-HMGA2 specific antibodies. A representative red ponceau stained membrane is shown as quantity and integrity control of the GST fusion proteins used.

As well as NPM1, HMGAs posses a modular organization. They have three highly conserved and positively charged DNA-binding domains (DBDs), a central protein-protein interaction domain (PID), and an acidic C-terminal tail (Fig. 3). We previously demonstrated by farwestern analyses that the region corresponding to the protein-protein interaction domain of HMGA1 and HMGA2 is necessary for NPM1 binding^19^,^20^. We performed mapping experiments using HMGA1a protein employing both C- and N-terminal deletion mutants in order to better clarify the domains used by HMGA1 proteins to contact NPM1 (Fig. 3a). Results summarized in Fig. 3b show that the truncated protein HMGA1a 1–79 retaining this region is still able to bind to NPM1, whereas the truncated form in which this region is deleted (1–51) is no longer able to perform it, similarly to what already reported^18^,^19^. Importantly, by using HMGA1a N-terminal deletion mutants, we demonstrated that the N-terminal HMGA1a portion is fundamental for interacting with NPM1, as well.

Interestingly, the interacting regions have opposite charges. The region of NPM1 is highly negative and that of HMGAs is highly positive, thus suggesting that NPM1/HMGA contact has an electrostatic contribution. To test this hypothesis, pull-down experiments were performed using full-length GST-NPM1 and both HMGA1a and HMGA2 proteins with increasing ionic strength. As shown in Fig. 3c, salt concentration above 150 mM completely abolishes the interaction, thus suggesting that the histone binding acidic clusters of NPM1 are involved in the contact with HMGA proteins. Plasmid DNA used to *in vitro* translate NPM1 is present in GST pull-down experiments. Since both HMGAs and NPM1 are DNA binding proteins^1^,^28^, to assess whether the detected interaction between these two proteins was not mediated by DNA, we performed GST pull-down experiments using increasing concentrations of EtBr, which has been shown to disrupt DNA-dependent protein-protein contacts^29^. As shown in supplementary Fig. S3, both HMGA1b and HMGA2 bind to NPM1 in the presence of EtBr, thus demonstrating that DNA does not mediate this interaction. The increment of HMGA/NPM1 binding affinity that we observed with increasing EtBr concentrations is probably caused by the change in the dielectric constant of the binding buffer due to the presence of EtBr itself. A direct HMGA/NPM1 interaction was further confirmed by GST-pull down experiments performed in the presence of DNase I (supplementary Fig. S4).

### NPM1 hampers HMGA/DNA binding

Histone chaperones aid the process of histone removal/deposition and constitute temporary reservoir for free histones^30^,^31^. In addition to histones^27^, NPM1 is able to bind to other nuclear basic proteins, modulating their DNA-binding activities^32^. Therefore, we investigated a possible role of NPM1 on HMGA-DNA binding properties by electrophoretic mobility shift assay (EMSA). Two different DNA probes were used, HCRII and E3, which are both recognized by HMGAs and correspond to the regulatory regions of *HOXD9* and *INSR* genes, respectively, whose activity is modulated by HMGAs^2^,^23^,^33^.

**Figure 4:**
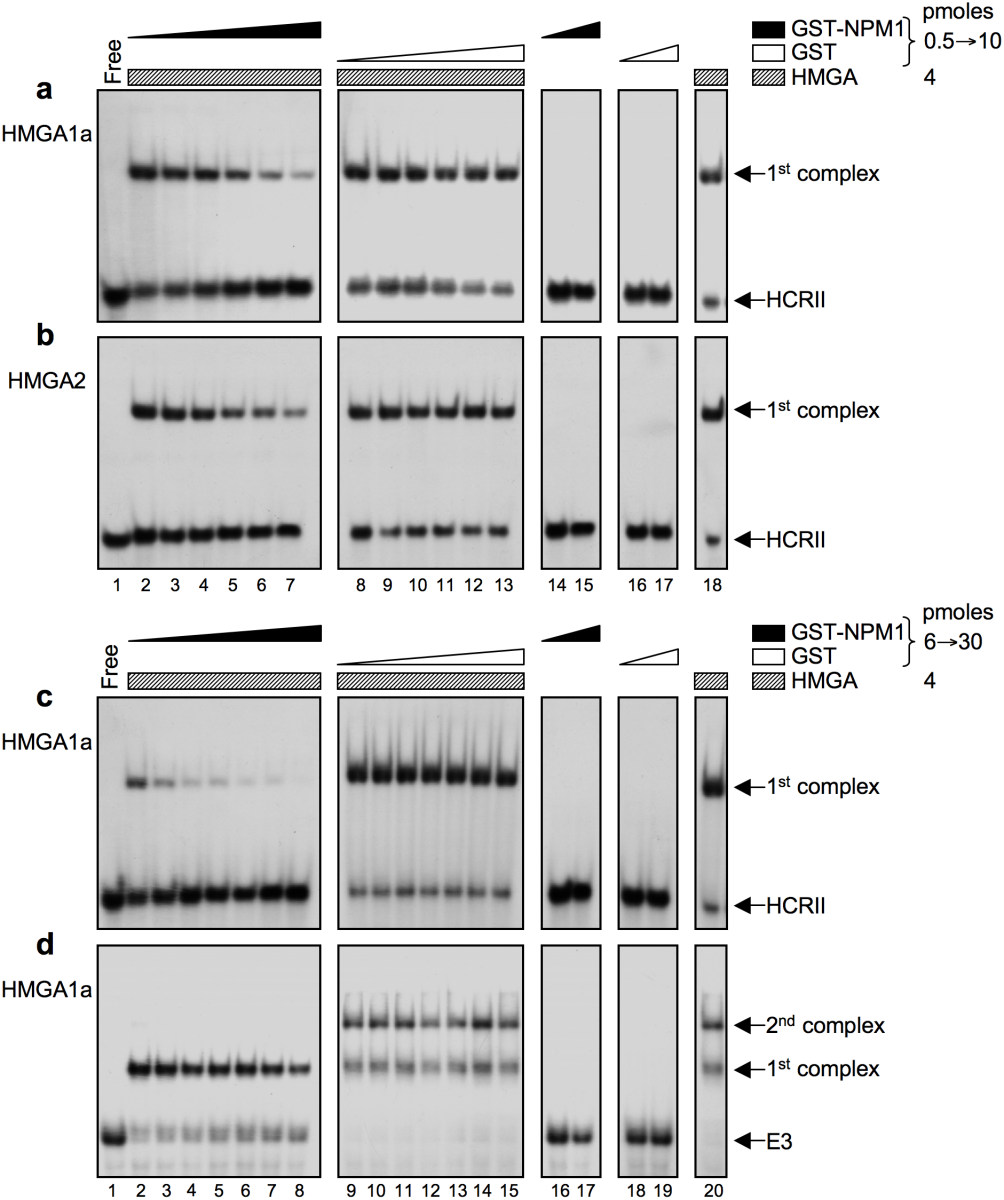
NPM1 modulates HMGA/DNA complexes formation. (a,b) Comparison of HMGA1a and HMGA2 binding to the HCRII oligonucleotide. (c,d) Comparison of HMGA1a binding to the HCRII and E3 oligonucleotides. HCRII: DNA sequence belonging to the HOXD9 gene promoter. E3: DNA sequence belonging to the INSR gene promoter. EMSAs were performed with constant amounts of HMGA proteins (4 pmoles) and [γ-^32^P] radiolabelled DNA probes (0.1 pmoles) and increasing amounts of GST-NPM1 or GST. 0.5, 1, 2, 4, 6, and 10 pmoles of GST-NPM1 or GST were used in EMSAs reported in panels a and b (lanes 2-7 and 8–13, respectively). 6, 10, 14, 18, 22, 26, and 30 pmoles were used in those reported in panels c and d (lanes 2–8 and 9–15). EMSAs performed with DNA probes and GST-NPM1 or GST (panels A and B, lanes 14–15 and 16–17: 0.5 and 10 pmoles, respectively; panels C and D, lanes 16–17 and 18–19: 6 and 30 pmoles, respectively) constitute negative controls whereas those with DNA probes and HMGA proteins alone are positive controls (panels a and b, lane 18; panels c and d, lane 20). The DNA probe alone is shown in panels a–d, lane 1. Results were visualized by autoradiography. Panels a, b, c, and d show images grouped from the same gel and therefore they have the same exposure time.

Figures 4a and b show EMSA experiments performed using the HCRII probe with fixed amounts of HMGA proteins and increasing quantities of GST-NPM1, while GST alone was used as a negative control. The presence of GST-NPM1 (from 0.5 to 10 pmoles) leads to an evident decrease of HMGA1a-HCRII complex formation (Fig. 4a, lanes 2–7). Same results were obtained when HMGA2 was used (Fig. 4b), while no effects were detected with increasing amounts of GST alone (Fig. 4a and b, lanes 8–13). Both GST-NPM1 and GST were not able to bind this DNA probe (lanes 14–17). Figures 4c and d report the comparison of NPM1 effect on HMGA1 binding affinity towards HCRII and E3 probes. Differently from HCRII, E3 contains multiple binding sites for HMGA proteins; therefore, two or more complexes can be detected when E3 is incubated with increasing amounts of HMGAs^22^. Consistently with the results shown in panel a, a further increase of NPM1 concentration (6 to 30 pmoles) causes a dramatic decrease of HMGA1a-HCRII complex formation up to its disappearance (Fig. 4c, lane 8). Interestingly, NPM1 behaviour with respect to HMGA1a-E3 complex formation seems to be different. The presence of GST-NPM1, even at the lowest concentration (6 pmoles), completely abolishes the formation of the second complex, while it strongly promotes the formation of the first one (Fig. 4d, compare lane 2 with lanes 9 and 20). On the contrary, the first complex formation is inhibited only at the highest NPM1 concentrations (Fig. 4d, lane 8). These results demonstrate that NPM1 modulates the binding of HMGA proteins to different DNA sequences. Since GST-NPM1 is unable to bind any of the DNA probes used, it is reasonable to assume that this effect is dependent on a direct interaction between HMGA and NPM1.

### NPM1 modulates the level of HMGA1 at the endogenous regulatory sequences of INSR and IGFBP1 genes

**Figure 5:**
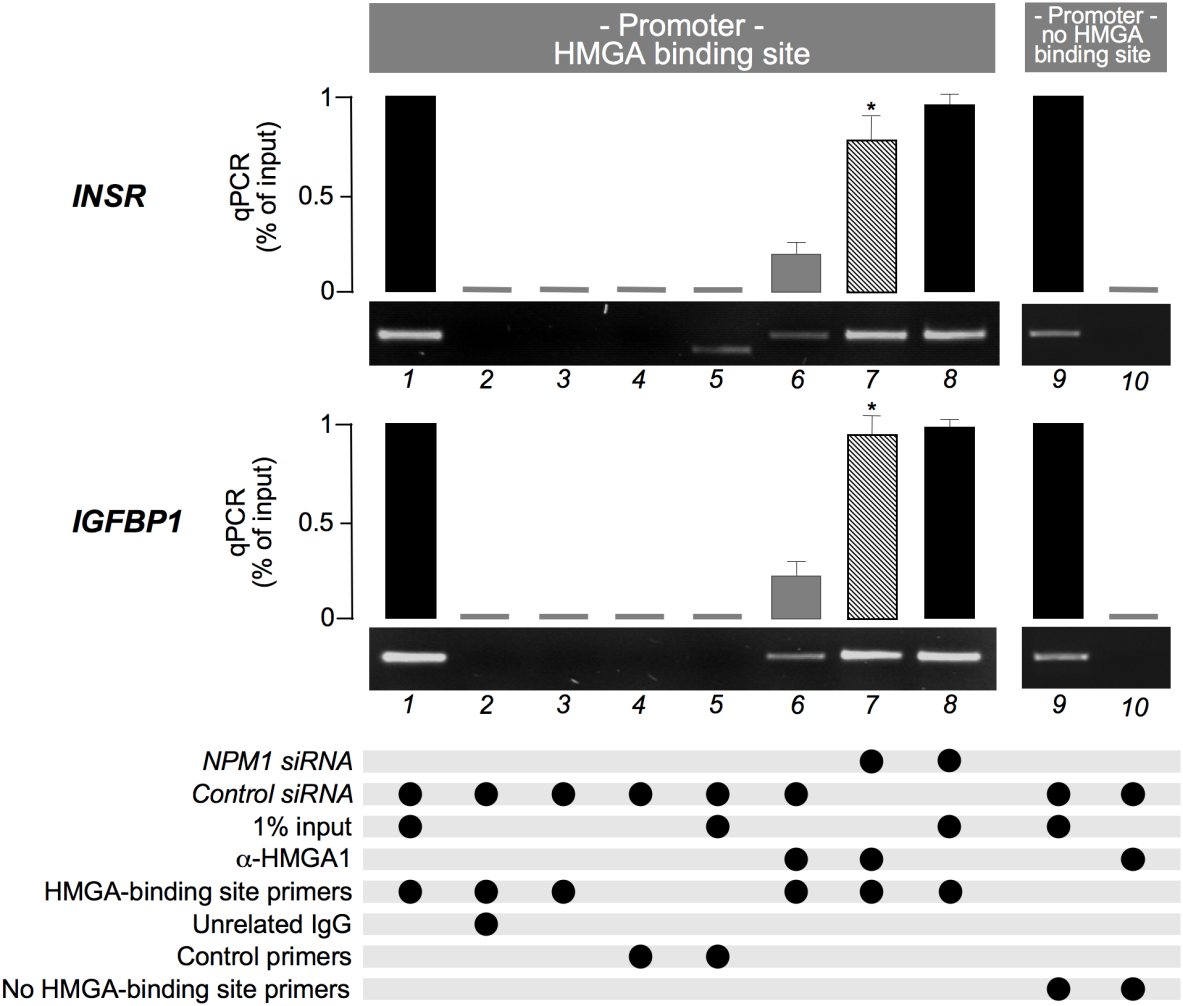
NPM1 modulates the *in vivo* INSR and IGFBP1 gene promoter occupancy of HMGA1. ChIP analyses for the evaluation of HMGA1 occupancy at the level of INSR (upper panel) and IGFBP1 (lower panel) promoters. Both qPCR data (histograms) and representative electrophoretic analyses of PCR-amplified immunoprecipitated DNAs are shown. ChIP analyses were performed in HepG2 cells pretreated with *NPM1* siRNA (lane 7) or control siRNA (lane 6), using an α-HMGA1 specific antibody. The relative quantity of promoter enriched by ChIP (HMGA1 binding region) was quantified by qPCR and expressed as a percentage of the input DNA (lanes 1 and 8, in both panels). On the right side of the panels (lanes 9 and 10), ChIP analyses evaluating no-HMGA1-binding-site regions at the level of INSR and IGFBP1 promoters are shown as negative controls. Lanes 2, 3, 4, and 5 show specificity controls for ChIPs. Lane 1 and 8 show input DNA from HepG2 cells treated with control siRNA or *NPM1* siRNA, respectively. All analyses were performed in triplicate and data are presented as mean ± s.e.m. *P < 0. 05 versus control siRNA treated cells (lane 6).

Next, we tested whether the NPM1 activity obtained *in vitro* towards HMGA1 could also be extended *in vivo*. In particular, we evaluated HMGA1 binding to the endogenous E3 region of the *INSR* gene promoter upon NPM1 depletion in HepG2 cells. A similar study was carried out using the promoter region of the *IGFBP1* gene, which, as we demonstrated before^4^ in this cell line, is also regulated by HMGA1. NPM1 expression was silenced in HepG2 cells using sequence-specific siRNA and the *in vivo* binding of HMGA1 to both INSR and IGFBP1 promoter regions was detected by ChIP, using an α-HMGA1 specific antibody. As shown in Fig. 5, binding of HMGA1 to the endogenous INSR and IGFBP1 loci was significantly increased in HepG2 cells exposed to siRNA against NPM1, thus indicating that NPM1 modulates HMGA1 binding to its target DNA in living cells.

### NPM1 modulates the activity of INSR and IGFBP1 regulatory sequences and their endogenous gene expression levels

**Figure 6:**
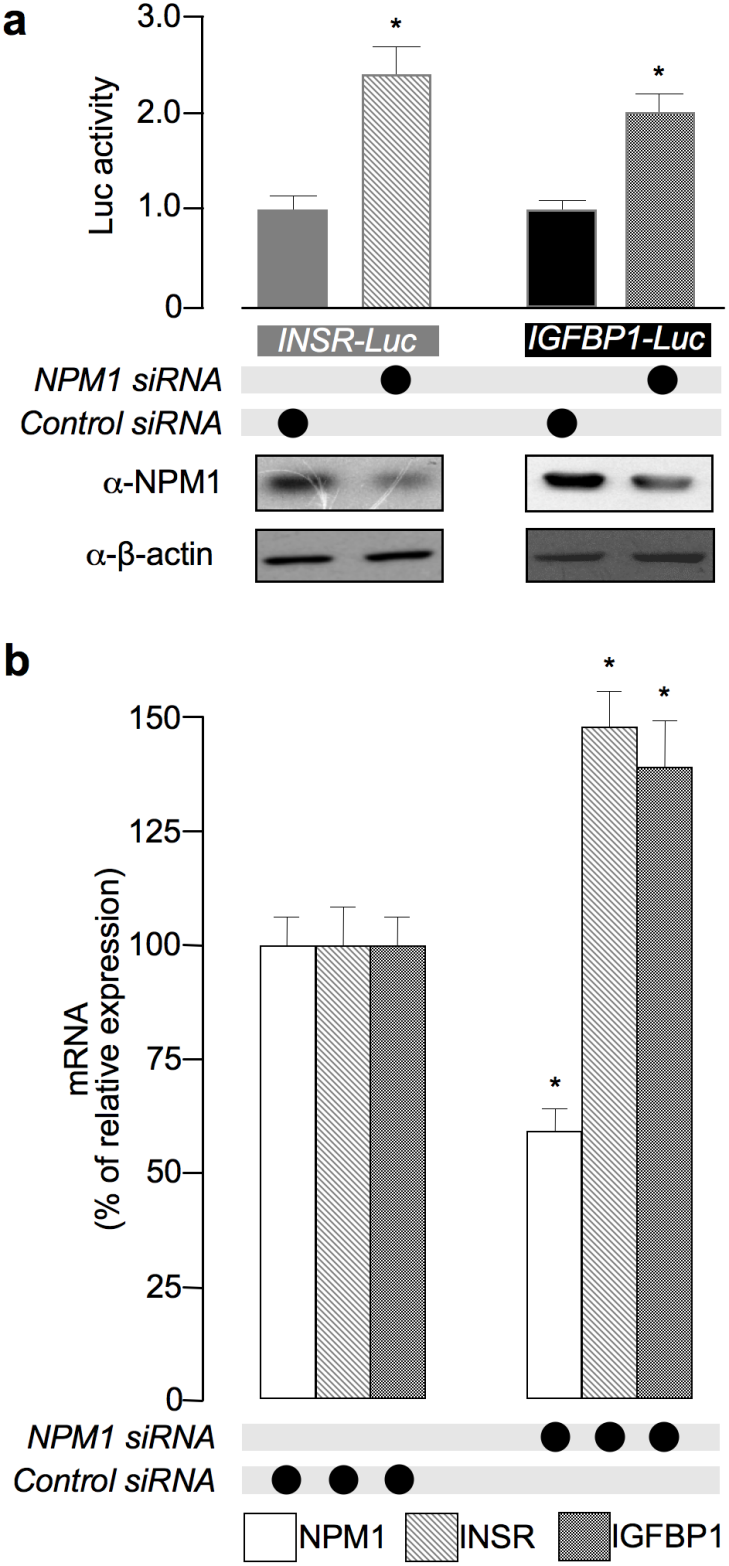
NPM1 modulates INSR and IGFBP1 gene promoter activities and their *in vivo* transcriptional outputs. (a) HepG2 cells, pretreated with *NPM1* siRNA or control siRNA, were transfected with luciferase vectors containing the promoter regions of either *INSR* (*INSR*-Luc) or *IGFBP1* (*IGFBP1*-Luc). Values are expressed as factors by which induced activity increased above the level of luciferase activity obtained in control siRNA transfections with either *INSR*-Luc or *IGFBP1*-Luc reporter, which is assigned an arbitrary value of 1. Western blot analyses of NPM1 in each condition are shown. β-actin, control of protein loading. Data are represented as mean ± s.e.m. from three independent experiments (*P < 0. 05 versus cells treated with control siRNA). (b) qRT-PCR analyses of *INSR* and *IGFBP1* expression in HepG2 cells transiently transfected with NPM1 siRNA or control siRNA after 72 hours. NPM1 mRNA expression level is shown as a control for NPM1 silencing efficacy. Data are represented as mean ± s.e.m. from three independent experiments (*P < 0. 05 versus cells treated with control siRNA).

As stated above, the binding of HMGA1 at the INSR and IGFBP1 promoters has been associated with the transcriptional activation of these two genes^2^,^4^. To see whether NPM1 had any functional effect on the INSR and IGFBP1 genes, we transfected HepG2 cells with luciferase reporter vectors harbouring the promoter region of either INSR or IGFBP1, and promoter activity was assessed as luciferase activity in cells, either silenced or not for NPM1 expression. Consistently with data from ChIP showing the increase of HMGA1 occupancy on both INSR and IGFBP1 gene promoters following cellular depletion of NPM1, the reporter gene activity of the INSR and IGFBP1 promoters was significantly increased in HepG2 cells that underwent NPM1 silencing (Fig. 6a), and this increase paralleled the increase in INSR and IGFBP1 mRNA levels (Fig. 6b), documenting a role of NPM1 in this scenario.

## Discussion

In this study we provide evidence for a new mechanism underlying the regulation of HMGA proteins activity, in which the molecular chaperone NPM1, by binding HMGA protein species, finely modulates HMGA/DNA complex formation at the level of enhancer-promoter sequences of target genes, hence affecting HMGA proteins function and therefore their transcriptional activity. NPM1 is a histone chaperone, which is involved at several levels in the process of chromatin opening and nucleosome depletion during transcriptional activation. NPM1 has been found to remove histone H1 from nucleosomal DNA^34^ and to disassemble acetylated nucleosomes^35^. Interestingly, NPM1 is also involved in mediating the proper deposition of histones and basic proteins in general onto DNA, thus preventing the formation of non-specific protein-DNA aggregation^27^,^31^,^32^. In addition, NPM1 has been demonstrated to be involved in the modulation of the activity of several transcription factors, such as the Interferon regulatory factor-1 (IRF-1) and the transcription factor Yin Yang 1 (YY1), whose DNA-binding and transcriptional activities are alleviated by NPM1^36^,^37^.

Herein we evaluated the effect of NPM1, on both *INSR* and *IGFBP1* promoters, clearly demonstrating that a decrease in NPM1 abundance is followed by an increase in the occupancy of promoter DNA by HMGA1, resulting in an increase in promoter activity. Consistently with these results, endogenous levels of both INSR and IGFBP1 mRNAs were significantly upregulated upon siRNA depletion of NPM1. Interestingly, EMSA experiments showed not only a negative modulatory role of NPM1 towards HMGA-DNA binding, but they also evidenced that the presence of NPM1 can selectively favor the formation of certain types of complexes with respect to others, what represents a peculiarity of the histone chaperone protein family, which depends on chaperone concentration. Indeed, as reported in the literature, DNA-binding specificity of basic proteins (e.g. histone H1) is regulated by the abundance of NPM1, which usually acts as a molecular chaperone driving the formation of specific protein-DNA complexes. However, above a certain threshold, NPM1 acts by sequestering basic proteins, thus preventing their binding to DNA^34^. This is in line with the concept that NPM1 can manifest opposite functional activities, depending on its expression levels.

**Figure 7:**
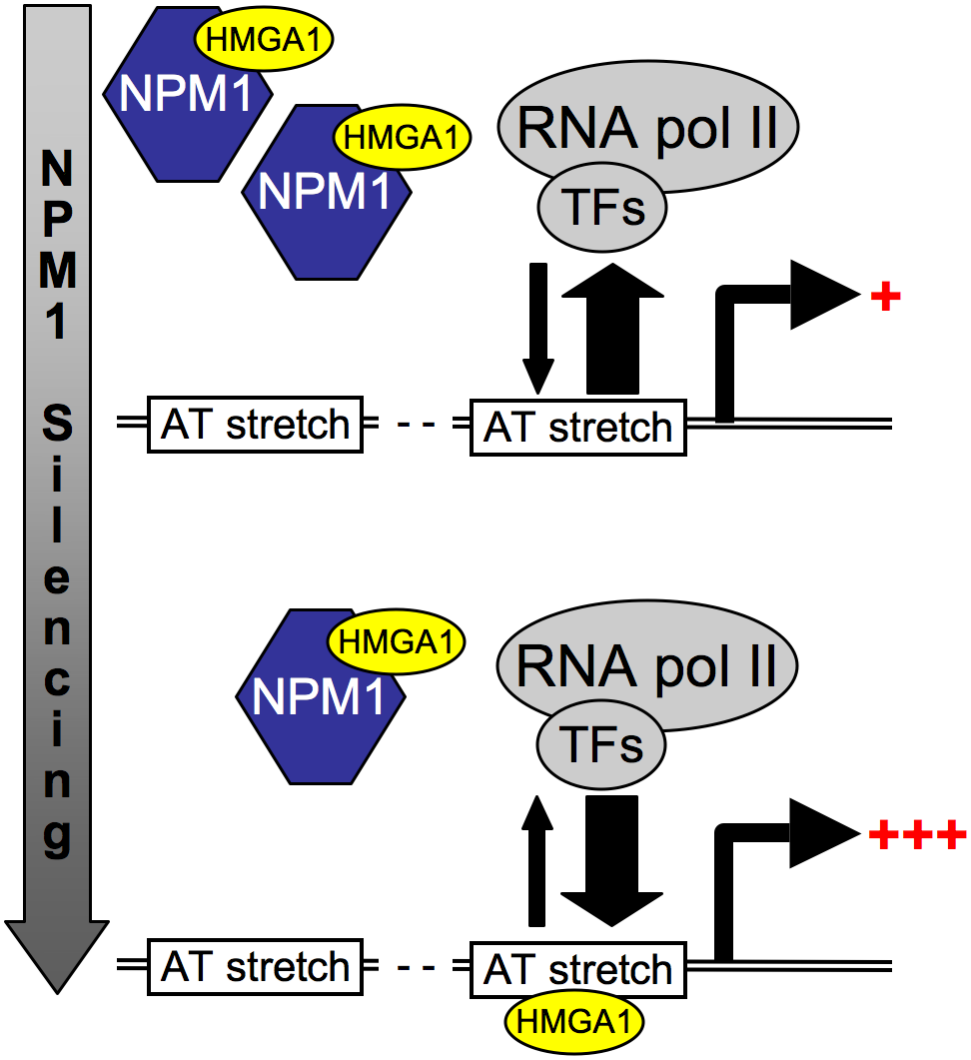
A model for the NPM1 modulatory role towards HMGA1. By directly binding to HMGA1 proteins, NPM1 could modulate the occupancy and binding specificity of these transcriptional architectural factors with respect to AT-stretches at the level of promoters/enhancers.

Overall, our results in the present study, must be interpreted taking into account at least three possible chaperoning roles of NPM1: towards (i) HMGAs, (ii) core histones, and (iii) the linker histone H1^27^,^31^,^32^,^34^,^35^. Indeed, H1 and nucleosomes pose a steric hindrance to the soft-landing of transcription factors on their own DNA binding sites, having to be removed for the activation of gene transcription^38^. Only when DNA is freely accessible, HMGA proteins can bind directly to the minor groove of AT-rich DNA stretches and organize the assembly of stereospecific nucleoprotein complexes, which are essential for correct gene expression^5^,^6^. Therefore, it is reasonable to assume that a triple contribution of NPM1 can be imagined in the context of INSR and IGFBP1 expression, as well as in the context of other HMGA-modulated genes. Based on these considerations and our results, it may be tempting to consider a model on the modulatory role of NPM1 towards HMGA target genes. When NPM1 is normally expressed, the formation of nucleosomes and higher order chromatin structures driven by H1 is favoured. Conversely, when NPM1 is overexpressed, such as in cancer cells, histones could be removed from DNA, leading to an open chromatin conformation. At this point, NPM1 could act towards HMGA proteins, whose binding affinity and specificity is also regulated by NPM1 (Fig. 7). Consistently with our results from EMSAs, below a certain expression threshold, NPM1 could selectively drive the formation of specific complexes, whereas above a certain expression threshold, NPM1 could sequester HMGA1. This view is supported by the fact that when NPM1 expression is downregulated by siRNA, the HMGA1 occupancy at the endogenous *INSR* and *IGFBP1* loci is increased and this increase is paralleled by an increase in the transcription rate of these genes.

In this work, we evaluated the interplay that occurs between NPM1 and HMGA1 in modulating the regulation of two glucose homeostasis-related genes, such as the *INSR* and the *IGFBP1* genes. As we previously reported, HMGA1 plays an essential role in the transcriptional regulation of these two genes^2^,^23^, and individuals with defects in HMGA1 have increased susceptibility to type 2 diabetes mellitus^3^,^17^,^39^,^40^ and metabolic syndrome^41^, a cluster of individual disorders all predisposing to cardiovascular disease. On the other hand, the involvement of HMGA proteins in cancer is well known^1^, as it is known the involvement of both the INSR and IGFBP1 in cell proliferation and neoplastic transformation^42^,^43^. Interestingly, both NPM1 and HMGAs are highly expressed in tumor cells. Thus, abnormalities in the interplay of NPM1 with HMGA proteins, by affecting *INSR* and *IGFBP1* gene regulation, might play a role in these conditions.

In conclusion, our results demonstrate a new post-translational regulatory mechanism controlling HMGA activities. This observation might have relevance to the pathophysiology of insulin-resistant syndromes and other pathological conditions where HMGAs have been implicated.

## Methods

### Co-immunoprecipitation (Co-IP)

HepG2 cells were cultured in DMEM 10% fetal bovine serum, 2 mM L-glutamine, 100 U/mL penicillin, and 100 μg/mL streptomycin. Cells were grown at 37°C in humidified 5% CO_2_ incubator, collected under subconfluence conditions, harvested in PBS, and lysed in Co-IP lysis buffer (25 mM Tris/HCl Ph 8, 0.5% v/v NP40, 10% v/v Glycerol, and 125 mM NaCl) supplemented with 1 mM PMSF, 1 mM NaVO_3_, 5 mM NaF, 10 mM Na(C_3_H_7_COO), and protease inhibitors cocktail (Sigma). Lysates were left for 15 minutes at 4°C and then sonicated (six times: 10 seconds pulse with 20 seconds pause, 30% power – Branson Digital Sonifier 250). Cellular debris was eliminated by centrifugation (15000 g, 10 min). Protein concentration was estimated by the Bicinconinic Acid method. 1 mg of lysate was used to perform a single Co-IP in 1 mL final volume (Co-IP lysis buffer) using 30 μL of Sepharose G protein resin (GE-Healthcare). Equal amounts of α-HMGA1 or α-HMGA2 antibody and pre-immune serum were previously immobilized on the resin. Resins were incubated with Co-IP lysis buffer supplemented with 1 mg/mL BSA for 1 h at 4°C and washed three times with Co-IP lysis buffer. Resins and lysates were incubated for 3 h at 4°C. Unbound proteins were removed with 3 washes using Co-IP lysis buffer. After elimination of the washing buffer, proteins were eluted by SDS sample buffer (40 μL). 8 μL of the eluted proteins were used for western-blot analyses. Inputs were obtained by directly conditioning a part of cell lysate with SDS sample buffer (75 and 2.5 μg for the detection of HMGAs and NPM1, respectively). Inputs and Co-IP proteins were western-blot analyzed with standard procedures using α-HMGA1, α-HMGA2, and α-NPM1 antibodies. α-NPM1 antibody was a monoclonal antibody purchased from Abcam (# ab10530), whereas α-HMGA1 and α-HMGA2 antibodies were rabbit polyclonal antibodies obtained in our laboratory.

### GST pull-down and farwestern assays

Recombinant GST-fusion proteins and HMGA proteins were expressed and purified as already described^19^. Plasmid vectors encoding for GST-fusion NPM1 forms (1–295, 1–259, 1–186, 1–117, 187–295, and 260–295) were kindly provided by P.G. Pelicci. pGEX 4T3 GST-NPM1 117-186 was generated by PCR with pGEX 4T3 GST-NPM1 1-295 as a template and the following primers: for 5′-GGCTGTGAATTCAGTAGCTGTGGAGGAA-3′; rev 5′-TATCGGCTCGAGTCATCAAGCTTCCTCATCATC-3′. *In vitro* production and radiolabeling of NPM1, GST pull-down, and farwestern experiments were performed essentially as already described^19^.

### EMSAs

GST and GST-NPM1 1–295 were produced as described above and eluted from Glutathione Sepharose 4B resin (GE Healthcare) by 50 mM Tris/HCl pH 8, 10 mM GSH. The indicated amounts of GST and GST-NPM1 1–295 (0.5, 1, 2, 4, 6, and 10 or 6, 10, 14, 18, 22, 26, and 30 pmoles) were incubated with 4 pmoles of HMGA proteins in 25 mM Tris/HCl pH 8, 5 mM GSH for 10 min at 4°C in a final volume of 8 μL. Radiolabelled DNA (0.1 pmoles) was then added to the protein mixture for an additional 10 minutes incubation in 180 mM NaCl, 1 mM MgCl_2_, 0.01% w/v BSA, 8% v/v glycerol, 10 mM Tris/HCl pH 7.9 (20 μL final volume) prior to gel loading. EMSAs were carried out essentially as previously described^22^. DNA probes (only the upper strand sequences are shown): E3: 5′-AGAAAAACTCCATCTAAAAAAAAAAAAAAAAAAAAAAAAAAACA-3′; HCRII: 5′-GACACATTAATCTATAATCAAATA-3′.

### ChIP

hIP was performed in cultured HepG2 cells, pretreated with either SMART pool ON TARGET Plus NPM1 siRNA (Dharmacon) or control siRNA as described previously^4^. Sequence-specific primers for *IGFBP1* and *INSR* gene promoters used for PCR and qPCR amplification are listed in Supplementary Information.

### Plasmid transfections and Luciferase assay

Recombinant Luc reporter constructs containing either human INSR-E3^23^ or IGFBP1 promoter^4^ were transiently transfected into HepG2 cells, using LipofectAMINE 2000 reagent (Invitrogen Life Technology Corporation, Carlsbad, Calif. USA), in the presence of siRNA targeting human NPM1 (SMART pool ON TARGET Plus NPM1 siRNA - Dharmacon) or nonspecific siRNA with a similar GC content (ON-TARGETplus Non-targeting Pool - Dharmacon). After knockdown for 72 h, the cells were prepared for analysis and luciferase activity was assayed in a luminometer (Turner Biosystems), using the dual-luciferase reporter assay system (Promega, Madison, Wis., USA). Renilla control vector served as an internal control of transfection efficiency, together with measurements of protein expression levels.

### Quantitative Reverse Transcription PCR (qRT-PCR)

For qRT-PCR, total cellular RNA was extracted from cells using the RNAqueous-4PCR kit and subjected to DNase treatment (Ambion). RNA levels were normalized against 18S ribosomal RNA in each sample, and cDNAs were synthesized from 2 μg of total RNA using the RETROscript first strand synthesis kit (Ambion). Primers were designed according to sequences from the GenBank database: human IGFBP1 (NM_000596.2): for 5′-CATTCCATCCTTTGGGAC-3′; rev 5′-ATTCTTGTTGCAGTTTGGCAG-3′. human *INSR* (NM_000208.2) for 5′-TTTGGGAAATCACCAGCTTGGCAGAAC-3′; rev. 5′-AAAGCTGGGGTGCAGGTC GTCCTTG-3′. A real-time thermocycler (Eppendorf Mastercycler ep realplex ES) was used to perform quantitative PCR. In a 20 μL final volume, 0.5 μL of the cDNA solution was mixed with SYBR Green RealMasterMix (Eppendorf), and 0.3 μM each of sense and antisense primers. The mixture was used as a template for the amplification by the following protocol: a denaturing step at 95°C for 2 min, then an amplification and quantification program repeated for 45 cycles of 95°C for 15 s, 55°C for 25 s, and 68°C for 25 s, followed by the melting curve step. SYBR Green fluorescence was measured, and relative quantification was made against ribosomal protein S9 cDNA used as an internal standard.

### Statistics

Statistical significance was evaluated using a 2-tailed Student's *t* test. P < 0.05 was considered significant. All bar graph data shown represent mean ± s.e.m.

## Author Contributions

L.A. performed Co-IP, GST-pull down, farwestern, EMSA, and immunofluorescence analyses and participated in the analysis and discussion of the data and drafting of the manuscript; R.S. conceived the study, performed Co-IP, GST-pull down, farwestern, and EMSA analyses, participated in the analysis and discussion of the data, and wrote the paper. E.C. participated in the analysis and discussion of the data and drafting of the manuscript; S.I. and B.A. performed ChIP, luciferase, and qRT-PCR analyses; S.P. performed immunofluorescence analyses. S.P. and I.P. participated in the analysis and discussion of the data and drafting of the manuscript; A.B. participated in the analysis and discussion of the data and drafting of the manuscript and coordinated and supervised the work of E.C., S.I. and B.A., G.M. conceived, coordinated, and supervised the entire study, analysed the data, and wrote the paper. All authors discussed the results and commented on the paper.

## Supplementary Material

Supplementary Information Supplementary Information
