# Supplementary material for: A novel mechanism of post-translational modulation of HMGA functions by the histone chaperone nucleophosmin
Source: Sci Rep. 2015 Feb 25;5:8552. doi: 10.1038/srep08552 (PMC4339810; doi:10.1038/srep08552)
Supplement: Supplementary Information [file srep08552-s1.pdf]

A novel mechanism of post-translational modulation of HMGA functions by the histone chaperone nucleophosmin.

Laura Arnoldo[1,#], Riccardo Sgarra[1,#], Eusebio Chiefari[2], Stefania Iiritano[2], Biagio Arcidiacono[2], Silvia Pegoraro[1], Ilenia Pellarin[1], Antonio Brunetti[2], and Guidalberto Manfioletti[1,*]

[1] Department of Life Sciences, University of Trieste, Trieste, 34127, Italy

[2] Department of Health Sciences, University "Magna Græcia" of Catanzaro, Catanzaro, 88100, Italy.

[#] These authors contributed equally to this work.

[*] Correspondence and request for materials should be addressed to G.M. (manfiole@units.it)

SUPPLEMENTARY METHODS

Immunofluorescence analyses

HepG2 cells were grown on glass slides and fixed with PFA 4%. After permeabilization with 0.3% Triton/PBS and saturation in 0.5% BSA/PBS, cells were incubated for 1 hour at RT with primary antibodies diluted in 0.5% BSA/PBS. Secondary antibodies were applied for 1 hr at RT, and the cells then were stained with Hoechst. The α-NPM1 antibody was a monoclonal antibody purchased from Abcam (# ab10530). The α-HMGA1 antibody was a polyclonal antibody developed in our laboratory. The images were visualized by a Nikon Eclipse e800 microscope and acquired by Nikon ACT-1 software.

GST pull-down assays

GST pull-down experiments were performed using GST, GST-NPM1, and GST-NPM1 117-186 and recombinant HA-tagged HMGA1a and HMGA2 proteins essentially as already described (19). Bound HMGA proteins were detected by western-blot using an α-HA antibody (Sigma).

GST pull-down experiments in the presence of ethidium bromide were performed essentially as already described (19) using recombinant GST, GST-HMGA1b, and GST-HMGA2 and *in vitro* transcribed and translated NPM1.

GST pull down experiments in the presence of DNase I were performed essentially as already described (19) using recombinant GST and GST-NPM1 1-295 and recombinant HMGA1a and HMGA2 proteins in the presence of 8.5 U/mL DNase I (Invitrogen – cat. n°18068-015). The binding buffer was slightly modified accordingly to DNase I requirements (Binding buffer: 25 mM Hepes pH 7.9, 50 mM KCl, 2 mM $MgCl_2$, 1 mM DTT, 0,01% NP40). To check for the presence of DNA in the GST-pull down experiments, after the last washing step of the GST-pull down protocol, the Glutathione Sepharose™ 4B resins (carrying proteins and eventually also contaminating DNA) were incubated with a SDS-containing DNA loading buffer (1.6 mM TrisHCl pH 7.6, 0.005 Bromophenol blue, 0.005 Xylene cyanol FF, 10 % Glycerol, 10 mM EDTA, and 0.17% SDS), heated at 65 °C for 10 minutes to eliminate DNA-protein interactions, and chilled on ice. Equivalent amounts of samples with respect to the protein analyses have been loaded on a native TBE PAGE (T=10%). Increasing quantities (15, 30, 60, 120 ng) of a reference 100 bp DNA ladder (Quick-Load 100 bp DNA ladder – New England Biolabs Inc.) were loaded on the same gel. DNA was visualized by conventional Ethidium Bromide staining.

ChIP

Primers used in ChIP assays:

PCR	Sequence (5'–3')	Size	Accession n.
IGFBP-1 AT-rich for	CAGAAAGAGAAGCAATTCCG	720 bp	NC_000007.14

IGFBP-1 AT-rich rev	TACCAGCCAGACGCGAGCAA		
INSR AT-rich for	AGATCTGGCCATTGCACTC	377 bp	NC_000019.10
INSR AT-rich rev	ATGCCAGTTCTGGGGAGGTA		
IGFBP-1 noAT-rich for	AGTAGAGATGGGGTTTTGCC	264 bp	NC_000007.14
IGFBP-1 noAT-rich rev	GATAGCAATGCCTTCTTGTG		
INSR noAT-rich for	TCCCCTGCAAGCTTTCCCTC	383 bp	NC_000019.10
INSR noAT-rich rev	TACTGAGCGGAGGCCCTTGCGGT		

qPCR

IGFBP-1 AT-rich for	ACTGGACTTTAACTGAGGG	260 bp	NC_000007.14
IGFBP-1 AT-rich rev	ATGCTCGCTGGATGGGATG		
INSR AT-rich for	AGATCTGGCCATTGCACTC	240 bp	NC_000019.10
INSR AT-rich rev	AAACCTATTCCTGCCTCTGGG		
IGFBP-1 noAT-rich for	AGTAGAGATGGGGTTTTGCC	264 bp	NC_000007.14
IGFBP-1 noAT-rich rev	GATAGCAATGCCTTCTTGTG		
INSR noAT-rich for	TCCCCTGCAAGCTTTCCCTC	136 bp	NC_000019.10
INSR noAT-rich rev	TGCTCGGGCCCGTAAACAAC		

Supplementary Figure S1 - Arnoldo et al.

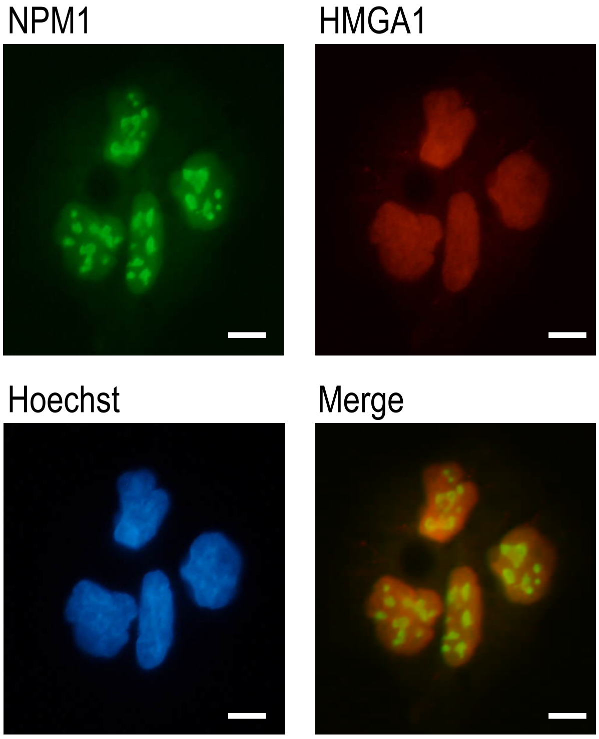

Supplementary Figure S1. HMGA1 and NPM1 co-localize in the nucleoplasm. HepG2 cells were fixed and double-immunostained for NPM1 (green) and HMGA1 (red). Nuclei were visualized by Hoechst counterstaining (blue). A merged image of HMGA1 and NPM1 is shown. Scale bar represents 10 μm.

Supplementary Figure S2 – Arnoldo et al.

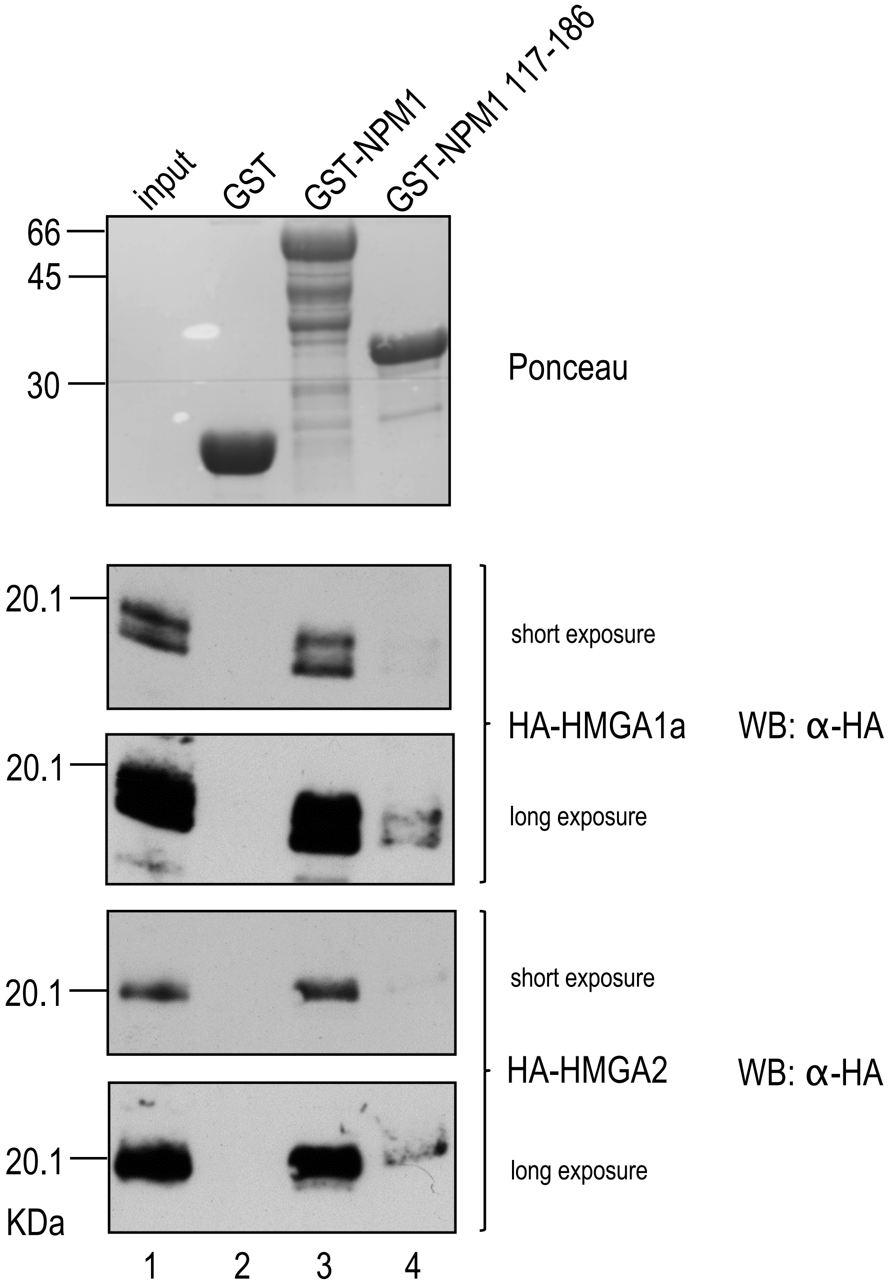

Supplementary Figure S2. NPM1 has almost the same binding affinity towards HMGA1a and HMGA2. Protein-protein interaction mapping experiment performed with GST pull-down assays using GST-fused NPM1 forms (full length 1-295 and 117-186) and recombinant HA-tagged HMGA1a and HMGA2 proteins. Bound proteins were visualized by western-blot using α-HA antibodies. GST was used as negative control. A representative red ponceau stained membrane is shown to verify the amount and integrity of the GST fusion proteins used. Two different exposure times (long and short) are shown.

Supplementary Figure S3 - Arnoldo et al.

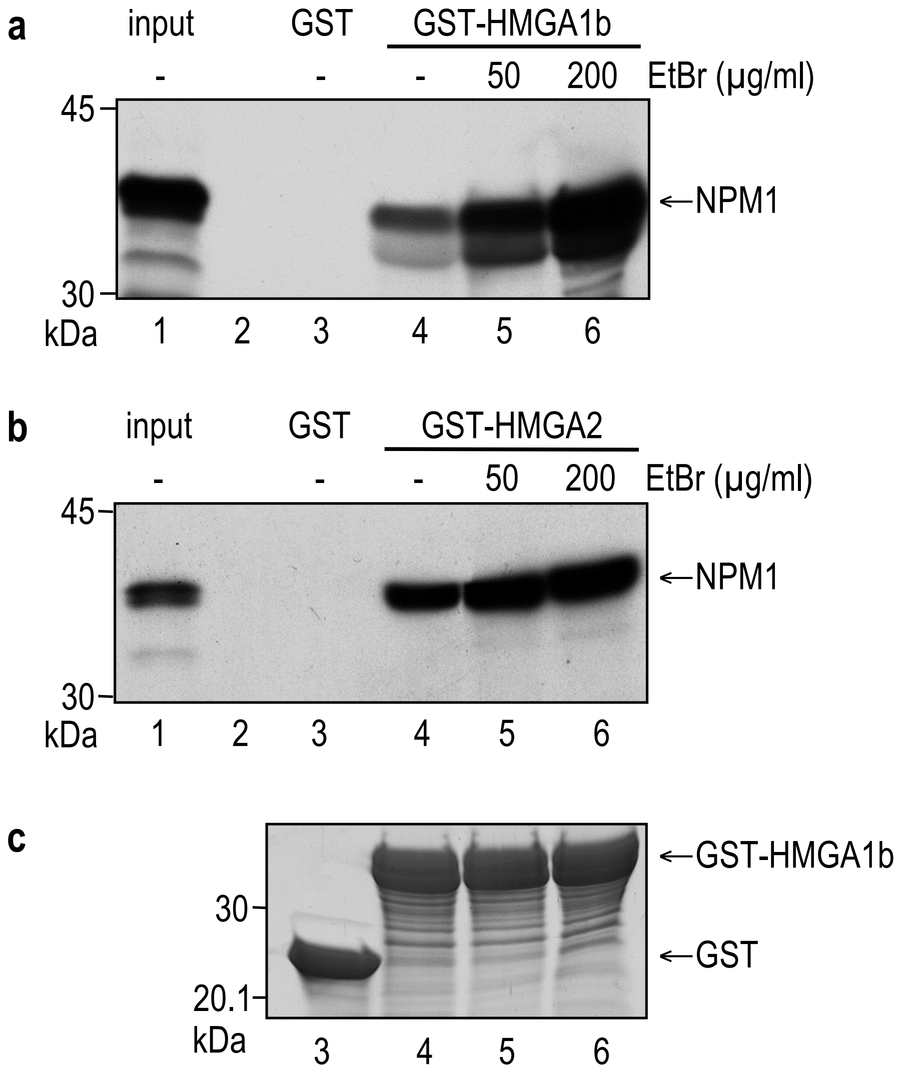

Supplementary Figure S3. HMGA/NPM1 binding is a direct interaction not mediated by DNA. GST pull-down experiments performed with GST-fused HMGA1b (a) and HMGA2 (b). NPM1 was produced by *in vitro* transcription and translation and [^{35}S]-radiolabelled. Increasing concentrations of ethidium bromide (EtBr) were used as indicated. Input is shown in lane 1 (i: 5% of the NPM1 amount used in GST-pull down). GST was used as negative control. Bound proteins were visualized by fluorography. (c) A representative Blue Coomassie stained gel to check for GST and GST-HMGA protein quantity normalization is shown.

Supplementary Figure S4 - Arnoldo et al.

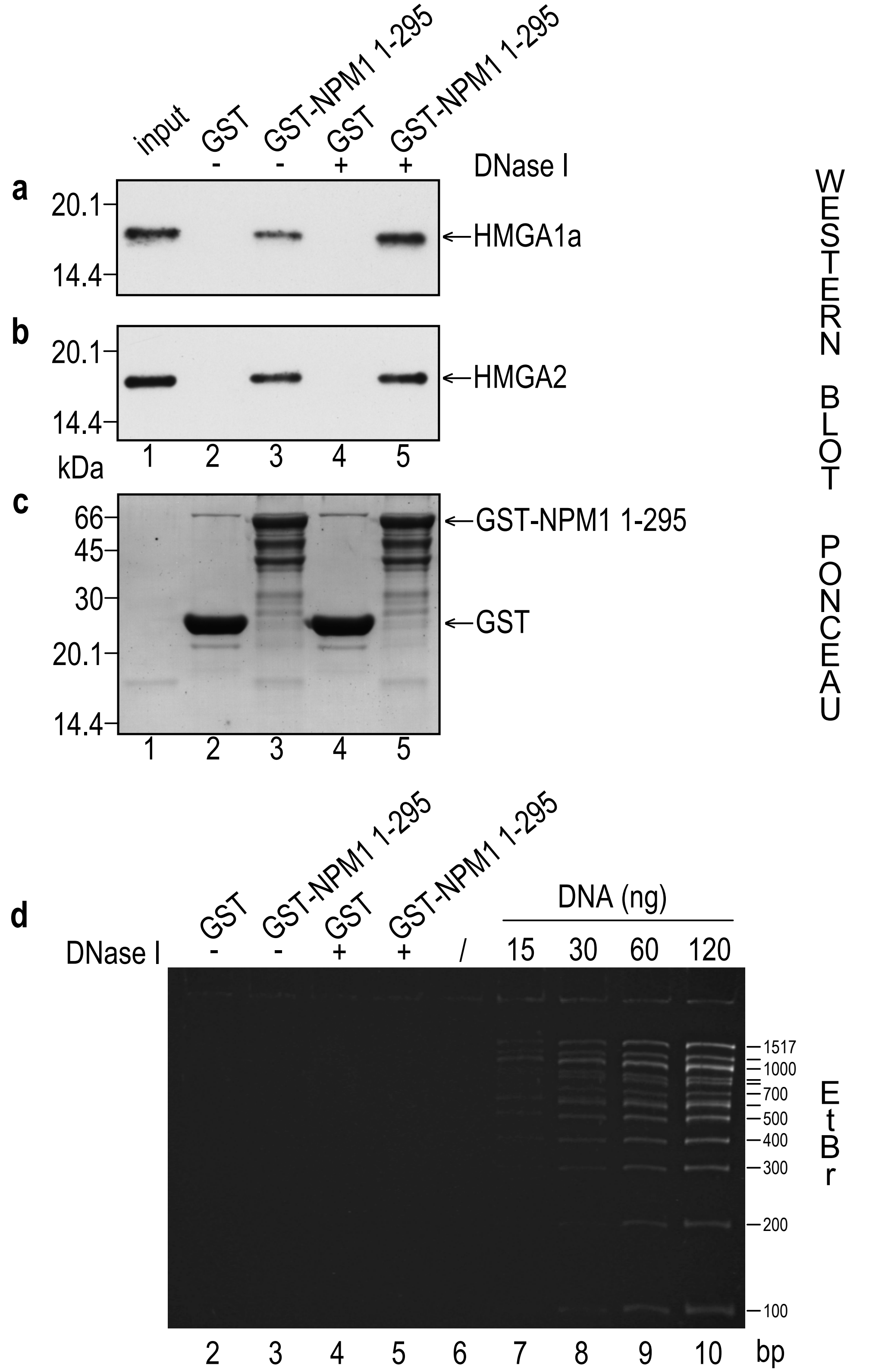

Supplementary Figure S4. HMGA/NPM1 binding is a direct interaction not mediated by DNA. GST pull-down experiments performed with recombinant HMGA1a (a) and HMGA2 (b). NPM1 1-295 was produced as a GST-fused protein and GST was used as a negative control. GST pull downs were performed in the presence (+) or absence (-) of DNase I (1.7 U/200 μL). Input is shown in lane 1 (i: 100% of the HMGA recombinant proteins amount used in GST-pull down - 0.1 μg). Bound proteins were visualized by western blot using α-HMGA1 or α-HMGA2 specific antibodies. (c) A representative ponceau stained nitrocellulose membrane to check for GST and GST-NPM1 1-295 protein quantity normalization is shown. (d) A representative Ethidium Bromide stained native TBE PAGE (T=10%) to check for the presence of DNA into GST-pull down assays (lanes 2-5). In lanes 7-10 different quantities of a 100 base pair DNA ladder are loaded (12 bands for a total of 15, 30, 60, and 120 ng; from the top the markers are: 1517, 1200, 1000, 900, 800, 700, 600, 500, 400, 300, 200, 100 bp).
